# Attitudes Towards Aging and Related Factors: A Cross-sectional Comparison of Gerontology and Other Undergraduates

**DOI:** 10.1093/geroni/igaf122.1664

**Published:** 2025-12-31

**Authors:** Shiyu Chen, Yongfang Tang

**Affiliations:** Hunan Women’s University, Changsha, Hunan, China (People’s Republic); Hunan Women’s University, Changsha, Hunan, China (People’s Republic)

## Abstract

Evaluating the young generation’s attitudes towards the older people is important to investigate on ageism, especially in higher education. This study aims to compare the attitudes towards older adults and aging between Chinese undergraduates from either an established gerontology program or other undergraduate programs, as well as to explore the influencing factors. 285 participants currently enrolled in Hunan Women’s University were recruited either from the gerontology program (n = 164) using purposive sampling, or other undergraduate programs(n = 121) using snowball sampling. The Kogan’s attitudes toward old people scale (KAOP), Anxiety about Aging Scale (AAS), and Aging Semantic Differential (ASD) were used to measure the students’ attitudes towards the older people and aging. ANCOVA analysis adjusted for sociodemographic covariates was used to examine the difference between majors. The KAOP score was significantly higher among gerontology students, compared to other students (152± 18.3 vs 146± 18.0, p < 0.01). The KAOP score was significantly associated with students’ current close relationship with older relative(s) (p < 0.05) and experience of growing up with older relative(s) (p < 0.05). Although AAS score were not different between programs, it was also significantly associated with students’ current close relationship with older relative(s) (p < 0.01) and experience of growing up with older relative(s) (p < 0.05). Our results suggest that gerontological higher education as well as previous intergenerational experience are beneficial in improving young adults’ attitudes towards aging and the older people, and further actions should be taken to enhance aging related education in a wider spectrum.

